# Mitochondrial Dysfunction in Rheumatoid Arthritis

**DOI:** 10.3390/biom12091216

**Published:** 2022-09-01

**Authors:** Chen Ma, Jie Wang, Fenfang Hong, Shulong Yang

**Affiliations:** 1 Experimental Center of Pathogen Biology, College of Medicine, Nanchang University, Nanchang 330006, China; 2 Queen Mary School, College of Medicine, Nanchang University, Nanchang 330006, China; 3 Department of Graduate, Shandong First Medical University, Shandong Academy of Medical Sciences, Jinan 250117, China; 4 Key Laboratory of Chronic Diseases, Fuzhou Medical College, Nanchang University, Fuzhou 344099, China; 5 Department of Physiology, Fuzhou Medical College, Nanchang University, Fuzhou 344099, China

**Keywords:** mitochondria, rheumatoid arthritis

## Abstract

Rheumatoid arthritis, a chronic autoimmune disease with complex etiology, is characterized by excessive proliferation of synovial cells, massive production of inflammatory cells and cartilage destruction. Studies have shown that mitochondrial dysfunction plays an important role in promoting the occurrence of RA. Mitochondria with normal structure and function are essential for the normal survival of chondrocytes and synovial cells. Once mitochondrial function is destroyed, it will affect the survival, activation and differentiation of immune cells and non-immune cells involved in the pathogenesis of RA, thus leading to the occurrence of RA. However, the mechanism of mitochondrial dysfunction in RA remains unclear. This article reviews the method of mitochondrial dysfunction leading to RA, the effects of mitochondrial dysfunction on immune cells, the etiology of mitochondrial dysfunction in RA, and the pathology of mitochondrial dysfunction in RA. We also outline some drugs that can exert therapeutic effects on RA which are associated with modulating mitochondrial activity. The understanding and summary of mitochondrial dysfunction in RA may provide new research directions for pathological intervention and prevention of RA.

## 1. Introduction

Mitochondria is an organelle with a double membrane and it is very widely distributed, presenting in almost all cells of the body [1]. Regarding the origin of mitochondria, the endosymbiotic theory is widely accepted by researchers. In this theory mitochondria are said to be derived from the merger of bacteria with proto-eukaryotic cells [2]. Mitochondria are critical energy-supplying organelles whose primary function is to produce adenosine triphosphate (ATP). They provide energy to the cell through the tricarboxylic acid cycle (TCA cycle) and oxidative phosphorylation. However, there is no doubt that mitochondria have several other roles in the cell, such as regulation of calcium homeostasis, production of reactive oxygen species, cell proliferation and metabolism. All these functions are important for the normal life activities of cells [3]. Meanwhile, mitochondria are also important for immune cells, including T cells, macrophages, and neutrophils [4]. According to recent studies, mitochondrial damage may be related to the pathogenesis of rheumatoid arthritis [1].

Rheumatoid arthritis (RA) is a chronic inflammatory autoimmune disease that mainly affects synovial joints and causes a series of severe inflammatory reactions [5]. The etiology of RA is complex and still being explored. Although recent therapeutic breakthroughs have been made, there has been no cure for the disease until now. Co-epitopes of human leukocyte antigen DR (HLA-DR) alleles in major histocompatibility complex II (MHC-II) are important genetic factors in RA [6]. It has also been hypothesized that the onset of RA is associated with the dysregulation of immune signaling pathways. The patient’s autoantibodies incorrectly recognize and attack self-antigens, leading to a series of inflammatory responses in the joints [7]. Autoantibodies such as rheumatoid factor, anti-citrullinated protein antibodies (ACPA) and anti-carbamylated protein antibodies are present in high levels in the plasma of RA patients [8]. Moreover, RA affects approximately 0.1–2.0% of the global population, with a higher incidence in females than in males [9]. The main symptoms of RA are swelling and pain in the joints and, in severe cases, deformity of the joints. This can be accompanied by complications such as anemia, osteoporosis, cardiovascular disease, and lung disorders [10].

Mitochondrial dysfunction is a critical factor in developing autoimmune diseases, including RA. Mitochondria are involved in disease pathogenesis by acting on different signaling pathways through direct and indirect effects. In this review, we address three pathways by which mitochondrial dysfunction leads to RA. We also discuss the effects of mitochondrial dysfunction on immune cells in RA. Furthermore, we discuss the causes of mitochondrial dysfunction in RA, focusing on the pathological role of mitochondrial disorders in RA and explaining the impact of mitochondrial disorders on RA occurrence and pathogenesis. Finally, we list some drugs that play a role in the treatment of RA by modulating mitochondrial activity. Overall, we review the physiological functions and pathological roles of mitochondria in RA to promote the understanding of mitochondrial mechanisms and to emphasize the important role of mitochondria in RA.

## 2. Three Ways That Mitochondrial Dysfunction Leads to RA

In RA, the most severely damaged cells are chondrocytes and synovial cells. If the mitochondrial function in these two types of cells is disrupted, it will lead to the dysfunction of cell physiology, and then trigger RA. Chondrocytes are a mature cell type in cartilage. They have a significant role in the production and maintenance of the cartilage matrix, which is essential for the health of the human joint. At the same time, mitochondria are important in maintaining the normal function of chondrocytes and the stability of the vivo environment [3].The synovial membrane is found in the inner lining of the synovium and is composed of fibroblast-like synovial cells (FLS) and macrophage-like synovial cells (MLS) [11]. RA is generally accompanied by the development of synovitis. In RA, synovial cells are activated due to stimulation and FLS secretes cytokines (e.g., Interleukin-6) to initiate inflammation at the joint. Activated FLS are hyperproliferative and resistant to apoptosis, leading to an increase in their number and exacerbating the symptoms of arthritis [12]. Many studies have shown that mitochondrial dysfunction is associated with abnormalities in synovial cell function, including promotion of synovial cell inflammation, inhibition of synovial cell apoptosis, promotion of synovial cell invasiveness, and promotion of synovial cell proliferation [11].

### 2.1. Abnormal Energy Metabolism

**Figure 1:**
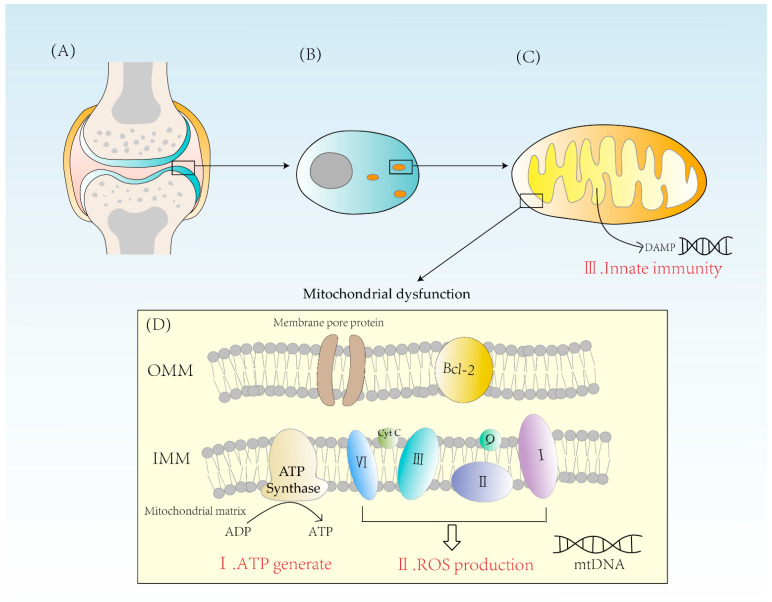
(**A**) A diagram of the articular cartilage structure. Cartilage is mainly composed of chondrocytes. (**B**) The distribution of mitochondria in chondrocytes is related to the energy requirement. (**C**) Mitochondrion is a double-membrane organelle. Mitochondria have two membranes, an outer membrane and an inner membrane. Mitochondrial inner membrane folds to form cristae. The mitochondrial matrix contains mtDNA. DAMPs activate innate immunity, causing inflammatory reactions. (**D**) Mitochondria synthesize ATP through ATPase and form ROS through complexes I, II, III, and IV. When mitochondria are dysfunctional, both excessive ROS and energy imbalance will contribute to the occurrence of RA. OMM = outer mitochondrial membrane, IMM = inner mitochondrial membrane, Bcl-2 = B-cell leukemia/lymphoma-2, DAMPs = damage-associated molecular patterns.

Mitochondria are indispensable for the energy supply of chondrocytes due to their essential physiological function of producing ATP from glucose. Glucose is converted to pyruvate in the cytoplasm through glycolysis. Then, pyruvate enters the mitochondrial matrix and is converted to acetyl coenzyme A which undergoes Krebs cycle and provides NADH for the electron transport chain [13]. The electron transport chain consists mainly of four enzyme complexes, coenzyme Q and cytochrome C, which transfer electrons and pump out hydrogen ions. The potential energy provided by the outflow of hydrogen ions powers the synthesis of ATP by ATPase. Because of the action of ATPase, one molecule of glucose can eventually produce 32 ATP [14]. Oxidative phosphorylation is of significant importance for cellular energy supply and is a highly efficient way of ATP synthesis (Figure 1).

If mitochondrial dysfunction occurs, the balance between glycolysis and oxidative phosphorylation is disrupted, affecting the synthesis of ATP, which in turn has irreversible effects on the production and metabolism of various substances. This is especially so for chondrocytes because, as the only mature cell in cartilage, if their ATP synthesis is reduced it will not only negatively affect the function of the chondrocyte itself but also have some impact on the extracellular protein synthesis and stability of the matrix. Meanwhile, ATP deficiency due to mitochondrial dysfunction may disrupt the repair of cartilage degradation [3].

### 2.2. Excess ROS Production

One of the reactive oxygen species (ROS) sources in chondrocytes is the mitochondria. The reactive oxygen species synthesized in mitochondria, also known as mtROS, is mainly generated due to proton leakage in the electron transport chain. In normal conditions, the production of one oxygen molecule requires the transfer of four electrons by the ETC after complex IV and four hydrogen ions are combined to form two H2O molecules (Figure 1) [15].

Though the electrons in the ETC leak during transfer, they will stay in unstable positions and are susceptible to oxidation by adjacent oxygen, producing ROS and hydroxyl (OH-) [16]. Under normal circumstances, about 2% of oxygen is used to produce reactive oxygen species (ROS), which has important physiological implications in the cell. A sufficient amount of ROS is required for chondrocyte repair and apoptosis, cytokine production, and extracellular matrix synthesis [17,18]. A rise in ROS levels leads to severe damage to cellular structures, including DNA, lipids, and proteins. Generally speaking, the mitochondrial antioxidant defense system can scavenge excess O2- and hydrogen peroxide to keep ROS levels low. At the same time, mitochondria can scavenge ROS produced by other cellular sources (e.g., macrophages) in order to protect cells from oxidative damage [3].

### 2.3. Activation of Innate Immunity

Mitochondria are essential organelles in the synthesis of ATP and control of programmed cell death in synovial cells, creating ROS [11]. They play an important role in energy supply, cell cycle control, and cell metabolism. Further, mitochondria also have a solid immunological function. Mitochondria are formed from internalized bacteria, according to the endosymbiotic theory. When mitochondria are damaged, damage-related molecular patterns (DAMPs) are released into the cytoplasm. These endogenous molecules can bind to corresponding receptors to elicit an immune response (Figure 1). For example, mtDNA released from mitochondria during mitochondrial damage is known as DAMP, which triggers immune responses and causes inflammation. Therefore, factors leading to mtDNA leakage may cause mitochondrial disorder, and eventually lead to the occurrence of inflammatory reactions, which may ultimately lead to RA [19]. DAMPs can activate innate immune cells and cause innate immune response to a certain extent. At present, a variety of DAMPs have been found, mainly including high-mobility histone B1, IL-1α, heat shock protein, etc. The process of inducing immune responses includes NF-κB signaling and induces the production of pro-inflammatory cytokines such as tumor necrosis factor-β (TNF-β) [20]. In this way, a strong innate immunity is activated, leading to the development of RA. 

## 3. Effects of Mitochondrial Dysfunction on Immune Cells in RA

### 3.1. T Cell

Under normal physiological conditions, primary T cells transform, after recognizing antigens on antigen-presenting cells (APCs), into effector T cells and begin to proliferate. Effector T cells have a mitochondrial oxidative metabolism dependency [1]. Glycolysis provides energy for the proliferation of effector T cells, as well as substrates for DNA and protein synthesis. Therefore, mitochondria play an important role in T cell proliferation and function [21].

**Figure 2:**
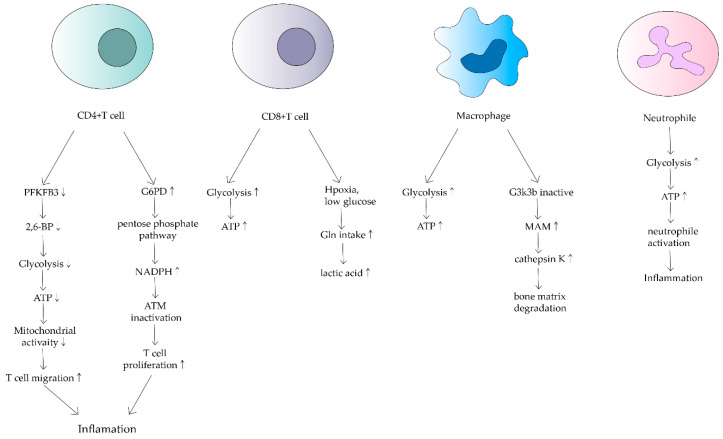
Effects of mitochondrial dysfunction on different immune cells in RA. This shows that ATP synthesis increases in CD4+ T cells, macrophages and neutrophils. In contrast, it decreases in CD8+ T cells. Different cells have different mechanisms that lead to inflammation and bone matrix degradation, causing the development of RA.

CD4+ T cells are the main cellular component of synovitis in RA and a key driver of pathogenic immunity. Yang Z et al. found that ATP levels, oxygen consumption and lactate production were reduced in CD4+ T cells of RA patients [22]. They suggested that in RA patients, 6-phosphofuran-2-kinase/fructose-2,6-bisphosphatase 3 (PFKFB3), an essential rate-limiting enzyme of the glycolytic pathway is inhibited. Under normal physiological conditions, PFKFB3 produces fructose 2,6-bisphosphate (a metabotropic activator of the glycolytic enzyme PFK-1), which contributes to glycolysis [23]. However, in CD4+ T cells in RA, induction of PFKFB3 is inhibited, resulting in a decreased glycolysis rate and a reduction in ATP synthesis [24]. Meanwhile, glucose-6-phosphate dehydrogenase (G6PD) expression is upregulated in CD4+ T cells. G6PD can catalyze the pentose phosphate pathway (PPP), causing NADPH and glutathione concentrations to increase (Figure 2). Besides, glutathione has an antioxidant effect. The enhanced antioxidant effect of excess glutathione prevents the activation of cell cycle checkpoint kinase ataxia telangiectasia-mutated (ATM), providing the possibility of T cell over proliferation. Meanwhile, overproliferating T cells are biased towards differentiation into inflammatory Th1 and Th17, exacerbating the response [25].

In addition to the adverse effects of reduced ATP production by oxidative phosphorylation in mitochondria on T cells, the TCA cycle of CD4+ T cells in RA can be disrupted by disruptions that increase levels of upstream metabolites such as α-ketoglutarate, citric acid and acetyl coenzyme A, leading to loss of granulomatous activity inline T cells [26]. This metabolic disruption enhances T-cell motility and migration, leading to greater inflammation and susceptibility to inflammation [27].

CD8+ T cells in RA synovium are also affected. However, in contrast to CD4+ T cells, CD8+ T cells in RA have been shown to increase the uptake of glutamine in hypoxic and low glucose conditions, resulting in lactate production increases(Figure 2) [28]. Alterations in both metabolic pathways of CD4+ T cells and CD8+ T cells can increase inflammatory mediators and are associated with RA development.

### 3.2. Macrophage

RA also alters the metabolic phenotype of macrophages, which is mainly related to the modulation of the normal activity of mitochondria. Glycolysis and oxidative phosphorylation are upregulated in macrophages due to pro-inflammatory requirements(Figure 2) [29]. In macrophages in RA patients, because of the upregulation of glycolysis and oxidative phosphorylation, more oxygen is consumed and more ATP is produced. Tight junctions with the endoplasmic reticulum are established to form mitochondria-associated membranes (MAMs) [30]. MAMs promote calcium transfer to maintain mitochondrial activity. In turn, the increased MAM association is dependent on the inactivation of glycogen synthase kinase 3b (GSK3b), which acts as a metabolic switch to regulate cellular respiration. In RA, most macrophages can inactivate GSK3b, allowing for increased MAM association, facilitating calcium transfer and increased ATP synthesis in mitochondria. In contrast, the formation of MAM and the persistence of GSK3b inactivation produces the collagenase cathepsin K, which makes it an important component of bone resorption and metabolism [31]. Cathepsin K destroys bone joints through the enhancement of bone resorption, something which is closely associated with the development of RA [32,33].

### 3.3. Neutrophil

Neutrophils are the source of stimulation and damage to extracellular mitochondrial DNA in autoimmune diseases. Mitochondrial dysregulation in neutrophils has an important impact on the course of RA [34]. Neutrophils are the origin of autoantigens which can promote inflammation and lead to tissue damage. The degree of neutrophil activity is heavily dependent on how much energy is produced by glycolysis. Researchers have found that neutrophils from RA synovial fluid exhibit enhanced glycolytic gene expression compared to peripheral blood neutrophils from other parts of the body (Figure 2). Mitochondria produce large amounts of ATP when they are dysfunctional, thus neutrophil activity is enhanced, leading to a series of inflammatory responses. Although a relevant role in RA has not been demonstrated, mitochondrial dysfunction has been shown to affect neutrophils, such as chemotaxis [34]. Because of the relatively few relevant studies, mitochondrial metabolism of neutrophils in RA deserves further investigation.

## 4. Etiology of Mitochondrial Dysfunction in RA

### 4.1. Hypoxia

**Figure 3:**
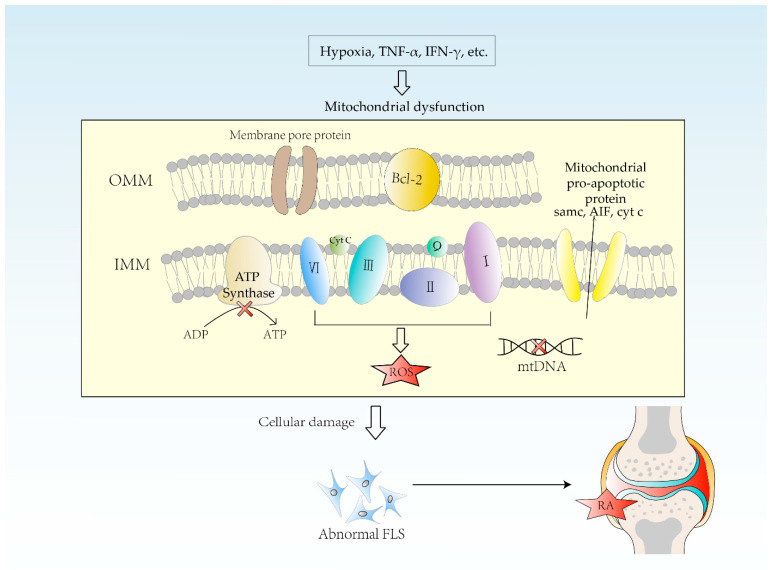
Etiology of mitochondrial dysfunction in RA. Hypoxia causes multiple changes in mitochondrial structure, genome, and dynamics, resulting in decreased ATP synthesis, excessive ROS production, and increased accumulation of mtDNA mutations. The more TNF-α and IFN-γ in synovium, the higher the frequency of mtDNA mutation. Mitochondrial dysfunction leads to cellular damage, including lots of inflammation reactions, which causes RA. FLS = fibroblast-like synoviocytes, OMM = outer mitochondrial membrane, IMM = inner mitochondrial membrane. Smac = second mitochondrial-derived activator of caspases, AIF = apoptosis-inducing factor, Cyt. c = cytochrome c.

Hypoxemia is defined as a state that has a below-normal level of oxygen in the blood. Hypoxia is associated with the pathogenesis of RA due to an enormous proliferation of synovial cells, vascular proliferation and leukocyte exudation (Figure 3). A hypoxic microenvironment can initiate inflammatory cell invasion mechanisms, which in turn accelerates cell proliferation and promotes migration [35]. The lower the synovial oxygen content, the more severe the synovitis. In the normal physiological state, there is a layer of endothelial cells arranged in the interior of the vessel. At this time, there is a little migration of leukocytes. The vasculature remains stable, and blood perfusion and oxygen supply are maintained at an average level. In contrast, there is incomplete interaction between the unstable blood vessels and the endothelial cells and pericytes in inflamed joints [36]. In inflammation, endothelial cells are activated, lose their polarity, detach and protrude into the vascular lumen, disrupting the pericyte layer, leading to vascular dysfunction. This restricts the transport of nutrients and oxygen, causing hypoxia [36]. Biniecka et al. cultured primary rheumatoid arthritis synovial fibroblasts (RASF) under hypoxic conditions, periodically testing their mitochondrial function. The results showed that hypoxia could induce mitochondrial dysfunction and promote glycolysis, abnormal vascularity and pannus production [37]. Besides, hypoxia can cause multiple changes in mitochondrial structure, genome, and kinetics, resulting in reduced ATP synthesis, excessive ROS production, and increased accumulation of mutations in mtDNA [38,39]. Understanding the mechanisms of hypoxia in RA may provide the basis for new therapeutic approaches.

### 4.2. mtDNA Mutation

The high mutation rate of mitochondrial DNA is explained by the fact that mitochondrial DNA is not protected by histone and chromatin structures and is easily exposed to damage caused by the environment. In addition, the activity of DNA repair enzymes in mitochondria is not as intense as that in the nucleus [40]. In general, mutations in genes encoding mitochondrial proteins result in altered peptide sequences, which leads to mitochondrial dysfunction. Mitochondrial dysfunction causes damage to the cell, such as increased ROS production, immune cell activation and autoantibody production. These can all exacerbate the course of RA (Figure 3) [35].

The inflammatory environment can promote mtDNA mutations. L. C. Harty et al. found that the more TNF-α or interferon Gamma (IFN-γ) in the synovium, the higher the frequency of mtDNA mutations. Treatment of RA FLS with TNF-α in vitro also resulted in an increased frequency of mtDNA mutations, suggesting that inflammation can lead to mtDNA mutations. There is a specific vicious cycle between mitochondrial dysfunction and inflammation [41]. Also, hypoxia is a key factor in induced mtDNA mutations. M. Biniecka et al. found that in RA fibroblasts cultured in a hypoxic environment in vitro the number of mtDNA mutations is increased. In RA patients, the lower the synovial oxygen partial pressure, the more random mtDNA mutations are present [42]. Specific mitochondrial haplotypes are also associated with RA. The major mtDNA variant loci related to RA are located within genes encoding ETC components. Mitsunaga S et al. found some rare single nucleotide variants associated with severe aggressive RA [43].

### 4.3. Oxidative Stress

Generally, excess ROS in mitochondria increases the risk of mtDNA mutations and ATP synthesis disorders and leads to mitochondrial dysfunction [18]. During the production of ATP, mitochondria produce waste products called reactive oxygen radicals. As mentioned above, ROS levels can increase dramatically due to certain environmental stresses (pathogenic factors) (Figure 3). Excessive accumulation of ROS causes oxidative stress, which may lead to DNA damage, making mtDNA encode for the wrong polypeptide, which damages the mitochondria themselves and also leads to cellular dysfunction and disease [15]. ROS has an important role in the pathogenesis of RA. ROS can also induce matrix degradation through mediators that directly or indirectly damage cellular components [44]. Hui Liu et al. found that nuclear receptor subfamily 1 group D member 1(NR1D1) could modulate joint inflammation and bone destruction in RA, reducing ROS production. They treated arthritis model mice with NR1D1 agonists and found that synovial proliferation, cellular infiltration, and cartilage destruction were inhibited [45]. Understanding the specific mechanisms of oxidative stress that lead to mitochondrial dysfunction may provide new directions for the treatment of RA.

## 5. Impact of Mitochondrial Dysfunction on RA

As previously mentioned, mitochondria are involved in many intracellular processes. They are central to metabolism and serve as important signaling hubs. The structural integrity of mitochondria is fundamental to proper cellular function. In recent years, there has been increasing evidence showing that mitochondrial structure and function changes can affect a range of physiological processes such as chondrocyte autophagy, induction of immune responses and disruption of mitochondria-related apoptotic pathways. Therefore, to provide a holistic understanding of these physiological processes, we summarize the link between changes in mitochondrial homeostasis and RA physiological processes from a pathological perspective.

### 5.1. Chondrocyte Autophagy

The structural integrity of mitochondria is considered a prerequisite for chondrocyte survival. Mitochondria have a regular oval shape under normal physiological conditions. They are involved in a variety of cellular physiological activities, such as energy supply, ROS production and regulation, mitochondria-mediated apoptosis, and calcium ion transport [37]. However, if mitochondria are damaged, the mitochondrial structure will change. These changes include a loss of folded cristae, which causes damage to the mitochondrial membrane and suggests a pathological state of the mitochondria [46].

Mitochondrial dysfunction may disrupt the balance between glycolysis and oxidative phosphorylation, resulting in a significant reduction in ATP production [47]. ROS are important for balancing cellular redox responses. Excess ROS activates multiple signaling pathways, and the activated signaling pathways promote cartilage degradation by inhibiting matrix synthesis, cell migration, and growth factor bioactivity. For example, by upregulating MMP leading to cartilage destruction, ROS impedes the action of growth factors on chondrocytes. These growth factors include the binding of chondrocytes to the extracellular matrix (ECM), ultimately leading to chondrocyte apoptosis [48,49].

Mitochondria-mediated autophagy is an important apoptotic pathway in chondrocytes. PINK1/Parkin is the best known autophagic pathway. Cell injury is accompanied by depolarization of mitochondrial membrane potential (ΔΨm), resulting in decreased ATP production as well as increased ROS, releasing pro-apoptotic cytokines such as Bcl-2 family members [50,51]. Besides, mitochondrial pro-apoptotic proteins like Cyto c, AIF and Smac are released. TNF-α and IL-1β are the two central cytokines involved in cartilage degradation, and they can affect the production of ΔΨm by decreasing the activity of MRC complex I [52]. Any error in the synthesis of ETC or ATP may lead to mitochondrial dysfunction and ultimately to dysfunctional chondrocyte physiology. As alluded to above, high concentrations of Ca^2+^ in mitochondria lead to cell death, while low concentrations of calcium ions lead to disruption of cellular energy metabolism. When cellular calcium homeostasis is disrupted, the ER moves Ca^2+^ to the mitochondria via the inositol 1,4,5-trisphosphate receptor (IP3R), which impairs mitochondrial function and triggers pro-apoptotic signals in chondrocytes [53].

In addition to Ca^2+^ imbalance, oxidative stress also accelerates apoptosis through the accumulation of advanced oxidation products (AOPPs), which are important markers in the RA patient. After oxidative damage, AOPPs are cross-linkers of protein side-chain amino acids. Increased ROS production, mitochondrial dysfunction and endoplasmic reticulum stress lead to activation of the cysteine family, causing apoptosis through activation of the endogenous apoptotic pathway in chondrocytes [54,55]. Mitochondrial dysfunction disrupts the repair activity against cartilage degradation and promotes the production of oxidative stress and inflammatory mediators. These pathological alterations further cause chondrocyte apoptosis, thereby enabling the development of RA.

### 5.2. Immune and Pro-Inflammatory Responses

Damaged mitochondria may lead to cellular dysfunction, immune cell activation and pro-inflammatory responses. In response to hypoxic stimuli, mitochondrial dysfunction alters cellular bioenergetics and promotes immune cells in an abnormally hypermetabolic state. When the integrity of the mitochondrial membrane is compromised and dysfunctional, components called damage-associated molecular patterns (DAMP) are released into the cytoplasm to induce inflammatory responses via pattern recognition receptors (PRRs) [56]. For example, during cell death or mitochondrial damage, mitochondria release endogenous oxidized mtDNA that is recognized as DAMP, which triggers an immune-mediated response. Any mitochondrial damage that results in the release of mtDNA from the mitochondrial matrix into the cytoplasm may interact with cGAS (cyclic GMP-AMP synthase) and lead to inflammatory responses [41].

The immune response promotes the release of inflammatory mediators, accumulating inflammatory cells, which increases oxidative stress. Oxidative stress causes normal mitochondria to become abnormal and creates a vicious cycle. We know that fluctuations in ROS levels are associated with mitochondrial dysfunction. In RA, microvascular disorders in synovial tissue can cause the creation of a hypoxic environment [57]. The proliferation of vascular opacities increases the energy demand for mitochondrial electron transport. Subsequently, mtROS production increases and further promotes glycolysis and inflammatory responses. Pro-inflammatory activity is mediated by HIF-1α, NF-κB, Janus kinase signaling and activator of transcription [35]. Additionally, mitochondrial dysfunction induces a low-grade inflammatory response in RA-associated cells and increases cellular sensitivity and expression of cytokine-induced inflammatory mediators. The occurrence of this process seems to be dependent on the production of ROS and the activation of NF-kB [32]. Mitochondria serve as important signal generators and signaling platforms used to activate NLRP3 and AIM2 inflammasomes. Mitochondrial dysfunction leads to activation of inflammasomes by decreasing oxidative metabolism and increasing mtROS production, which results in caspase-1 activation and increased IL-18, IL-1b release. The T-cell apoptosis that results from inflammatory vesicle triggering is a powerful stimulant of tissue inflammation [58].

### 5.3. Apoptosis Pathway Disorders

Activated FLS in RA is characterized by excessive proliferation and resistance to apoptosis [59]. Disruption of mitochondria-related apoptotic pathways affects apoptosis in synovial cells. Apoptosis resistance in synovial membranes is influenced by multiple factors. In general, alterations in mitochondrial function and structure promote resistance to apoptosis in synovial cells. Several cytokines such as IL-15, IL-6 and TL1A, and signaling pathways such as NF-κB are involved [60]. An important initiation signal for the mitochondrial-mediated apoptotic pathway is the increased permeability of the outer mitochondrial membrane, causing mitochondrial apoptotic factors Cyto c, AIF, and Smac entry into the cytoplasm, which ultimately leads to apoptosis. As a key regulator of apoptosis, Bcl-2 family proteins regulate the integrity of the mitochondrial outer membrane through the balance between pro-apoptotic factors (Bax, Bak and Bid) and anti-apoptotic factors (Bcl-2, Bcl-xL, A1 and Mcl-1) [61,62]. Enhanced expression of anti-apoptotic Bcl-2 family members plays a major role in developing inflammation in synoviocytes, which may also be responsible for anti-apoptotic cell death in synoviocytes. In addition, IL-15, a cytokine with anti-apoptotic properties, could also increase the expression of Bcl-xl and Bcl2, thus promoting apoptosis in synoviocytes [63]. Studies have confirmed that the IL-6/sIL-6R complex might inhibit apoptosis in FLS by increasing Bcl-2 expression and NF-κBactivation [64]. Similarly, TL1A/TNFR2-mediated mitochondrial dysfunction helps to make FLS more resistant to apoptosis by increasing Bcl-2 expression and downregulating the apoptotic factor caspase-8 [65]. A better understanding of the mechanisms of apoptotic pathways will help identify more precise treatments for RA patients and develop more effective therapies.

## 6. Drugs Associated with Mitochondria in RA Treatment

The main purpose of RA treatment is to reduce the inflammatory response of the joints and to inhibit lesion progression and irreversible bone destruction. A growing number of studies have shown that mitochondrial dysfunction is related to the pathology of many common diseases such as neurodegeneration, metabolic disorders, heart failure, ischemia-reperfusion injury and protozoal infection [66]. Some therapeutic strategies aimed at restoring mitochondrial function are emerging, and a few drugs have entered clinical trials. The direct or indirect effects of many of the drugs currently used to treat RA on the regulation of mitochondrial function are also becoming increasingly evident. On the one hand, these drugs contribute to anti-inflammatory and immunomodulation. On the other hand, they may also lead to side effects. These drugs, shown below, may not be designed to target mitochondria, but they have mitochondria involved in their mechanism of treatment. In other words, mitochondria are involved in the process by which drugs are metabolized in the body. It remains to be seen whether some drugs have therapeutic effects with regards to the improvement of mitochondrial function.

### 6.1. Conventional Synthetic Anti-Rheumatic Drugs (csDMARDs)

#### 6.1.1. Methotrexate

Methotrexate (MTX) is one of the most commonly used drugs to treat rheumatic diseases. MTX is structurally similar to folic acid, which inhibits the mitochondrial folate pathway by competitively inhibiting the folate-dependent enzyme, leading to a decrease in purine and pyrimidine synthesis and ultimately reducing cell proliferation [67]. In addition, MTX reduces oxidative stress by altering intracellular ROS levels and affecting mitochondrial membrane potential, which contributes to ROS production, thereby affecting cell function and survival [68]. Elevated ROS can propagate cellular oxidative stress, exerts inhibitory effects on monocytes and cytotoxic T cells, and induce T cell apoptosis, which contributes to the anti-inflammatory effects of the drug [69]. Lee et al. found that MTX can induce apoptosis in cultured synovial cells in a mitochondrial and caspase-dependent manner, which indirectly affects the inflammatory microenvironment [70]. However, mitochondrial dysfunction is also associated with adverse effects during MTX treatment. For example, MTX promotes platelet apoptosis through JNK-mediated mitochondrial damage and cytochrome c release, leading to liver injury. MTX nephrotoxicity is caused by mitochondrial membrane potential, decreased mitochondrial dehydrogenase activity, lipid peroxidation, and increased mitochondrial permeability [71,72,73].

#### 6.1.2. Leflunomide

Leflunomide (LEF) is a csDMARD for the treatment of RA in adults, with similar efficacy and adverse effects to MTX [74]. LEF inhibits the mitochondrial inner membrane protein dihydroorotate dehydrogenase (DHODH), which reduces the synthesis of pyrimidines, thereby inhibiting proliferation. It also produces anti-inflammatory effects by inhibiting tyrosine kinase activity and interrupting cellular inflammatory signaling. Recently, LEF has been shown to promote enhanced expression of mitochondrial fusion elements 1 and 2 in response to the stress of loss of pyrimidine synthesis, which leads to increased mitochondrial fusion and promotes mitochondrial elongation to confer stress resistance, enabling cells to resist death [75]. Leflunomide and its active metabolite, teriflunomide, also inhibit respiratory chain complex III activity to interfere with oxidative phosphorylation (OXPHOS) and aerobic glycolysis in activated T cells [76]. Inhibition of both DHODH and complex III by LEF increases ROS production, which induces apoptosis through oxidative stress [77]. Mitochondrial dysfunction is associated with liver injury, one of the serious adverse effects of LEF, which has potential mitochondrial sensitivity and leads to ATP depletion, LDH release and mitochondrial membrane depolarization in the hepatocellular carcinoma cell line HepG2, resulting in cytotoxicity [78].

#### 6.1.3. Sulfasalazine

Sulfasalazine is another csDMARD that is generally used in combination with other csDMARDs. Sulfasalazine inhibits purine synthesis in response to intestinal microbial action, causing increased adenosine release and binding to A2-type adenosine receptors on the surface of inflammatory cells to exert anti-inflammatory effects. Sulfasalazine can alter mitochondrial permeability and induce apoptosis in T cells. Sulfasalazine-induced apoptosis is partially mediated by apoptosis-inducing factor (AIF). Sulfasalazine causes mitochondrial nuclear translocation of AIF, and the accumulation of Bax mitochondria triggers AIF release to clear inflammatory cells and break the cycle of cell activation and tissue damage that continues in chronic inflammation [79]. In this way, it clears inflammatory cells and breaks the cycle of cell activation and tissue damage that persists in chronic inflammation. Renal injury induced by Sulfasalazine is also associated with mitochondrial disruption. Markers of oxidative stress, including increased ROS and lipid peroxidation (LPO), were detected in rat kidneys after dosing, which was associated with mitochondrial depolarization, GSH depletion and swelling [80]. In addition, some botanicals are used in RA treatment. Tanshinone IIA is extracted from the root of Salvia miltiorrhiza which has pro-apoptotic and anti-inflammatory activities. Tan IIA can regulate the protein expression of Bcl-2, Bax and Apaf-1, the release of mitochondrial Cyt-c and other mitochondrial pathways to induce apoptosis in RA-FLS cells [81]. Resveratrol (Res) is a non-flavonoid polyphenolic organic compound with multiple biological functions and activities. Res inhibits the production of mitochondrial ROS by activating the Nrf2 pathway, thereby inhibiting the proliferation and migration of RA-FLS and inducing apoptosis [82]. These drugs are effective in the treatment of RA, but the mechanism of action and therapeutic potential needs further study.

### 6.2. Biological Agents DMARDs (bDMARDs)

Biologics have been effective in treating RA patients who have not met the criteria for traditional anti-rheumatic drug therapy. In particular, it plays an important role in the treatment of refractory RA. Biological agents can block cytokine signaling, closely related to mitochondrial biology.

Tocilizumab is an IL-6 receptor antagonist. Oxidative stress was improved in patients treated with tocilizumab, suggesting that countering cytokine-induced ROS production may be an important therapeutic mechanism [83]. Infliximab and adalimumab are monoclonal antibodies against TNF. The use of TNF blocking therapies inhibits oxidative stress and hypoxia-induced mitochondrial mutations in inflammatory arthritis. These mitochondrial genomic alterations are rescued in patients who are clinically responsive to treatment [84]. An analysis of gene expression profiles in peripheral blood cells of RA patients after anti-TNF-α treatment found that ribosome and protein synthesis, immune response, redox, and mitochondrial electron transfer were the most affected pathways in PBMC during drug administration [85]. This suggests that modulation of mitochondrial activity plays a role in the therapeutic effects of a variety of biological drugs.

### 6.3. Targeted Synthetic DMARDs (tsDMARDs)

Recently, the research and application of targeted small molecule drugs have become a novel class of current therapeutic strategies for RA. JAK inhibitors have been successfully applied in clinical treatment. After several different cytokines bind to the receptor tyrosine kinase binding transmembrane receptor, the JAK tyrosine kinase family downstream of the cytokine receptor is able to dock and recruit the downstream signaling molecule STAT to send signals [86]. STAT3 activation has been previously associated with hypoxia and promoted the activity of HIF-1α [87]. Tofacitinib, the first JAK inhibitor approved by the FDA and EMA, significantly increases oxidative phosphorylation and ATP production in RA-FLS, resulting in a decrease in mitochondrial membrane potential, ROS production, and glycolysis-related genes and HIF-1α. Tofacitinib also differentially regulates key mitochondrial genes [88]. Inhibition of specific JAKs may block more than one pathway, explaining the efficacy and side effects observed with JAK inhibitors.

## 7. Summary and Perspectives 

Mitochondria dysfunction directly or indirectly affects the cellular microenvironment, leading to damage or overactivation of cells associated with RA pathogenesis. There are three ways that mitochondrial dysfunction leads to RA, including abnormal energy metabolism, excess ROS production and activation of innate immunity. T cells are affected by abnormal oxidative phosphorylation pathways, thereby exacerbating inflammation. Furthermore, the metabolic phenotype of macrophages is altered, disrupting bone metabolic homeostasis. However, research on mitochondrial dysfunction in other immune cells, such as neutrophils, are relatively lacking and deserve further exploration. Moreover, some factors like hypoxia, oxidative stress, and mtDNA mutation can lead to mitochondrial dysfunction from different perspectives. At the same time, mitochondrial dysfunction may also affect RA in different ways. DMARDs are the mainstream drugs for RA now. Further research into the relationship between mitochondrial dysfunction and RA may provide new ideas for the development of therapeutic methods targeting mitochondria in the future.

## Data Availability

Not applicable.
